# Midgut Bacterial Diversity of Wild Populations of *Phlebotomus (P.) papatasi*, the Vector of Zoonotic Cutaneous Leishmaniasis (ZCL) in Turkey

**DOI:** 10.1038/s41598-017-13948-2

**Published:** 2017-11-01

**Authors:** Mehmet Karakuş, Burçin Karabey, Şaban Orçun Kalkan, Güven Özdemir, Gizem Oğuz, Özge Erişöz Kasap, Bülent Alten, Seray Töz, Yusuf Özbel

**Affiliations:** 1 Department of Parasitology, Faculty of Medicine, Ege University, İzmir, Turkey; 2 Department of Biology, Faculty of Science, Ege University, İzmir, Turkey; 3 Department of Biology, Faculty of Science, Hacettepe University, Ankara, Turkey; 4 Present Address: Yale School of Public Health, Yale University, 60 College Street, New Haven, Connecticut USA

## Abstract

Phlebotomine sand flies are hematophagous insects that harbor bacterial, viral and parasitic agents like *Bartonella* sp*., Phleboviruses* and *Leishmania* spp., respectively. There are few reports on bacterial microbiota of *Phlebotomus (P.) papatasi* but no data available for natural populations of Turkey, where leishmaniasis is endemic. Therefore, we aimed to investigate the midgut bacterial flora of different populations of *P. papatasi*. Sand flies were collected from different towns (Karaburun, Urla, Ayvacik and Başçayır) located in the western part of Turkey. Laboratory reared *P. papatasi* were included in the study as an insectarium population. After sterile washing steps, sand flies were dissected and guts were separated. Three pools, (males, unfed females and blood-fed females) were generated for each population. Prokaryotic 16 S rRNA gene was amplified and DGGE was performed. Fourteen different organisms belonging to two Phylum (Proteobactericea and Furmicutes) were identified according to sequence results in the studied pools. The presence of *Wolbachia* sp. was shown for the first time in the wild-caught sand fly populations of Turkey. This is the first report of gut bacterial flora of wild-caught *P. papatasi* collected in an endemic area for leishmaniasis in Turkey. Microbiome profiling of wild-caught sand flies will be of great help in the investigating of possible vector control candidates for paratransgenic control approach.

## Introduction

Leishmaniasis is a neglected tropical disease caused by an intracellular protozoon parasite *Leishmania* and is transmitted by the bite of infected female Phlebotomine sand flies. According to the reports of WHO, the disease is endemic in 102 countries^1^. Phlebotomine sand flies are the vectors of leishmaniasis both in Old and New World^2^. Female sand flies are hematophagous and blood-feeding on different mammal hosts in order to develop eggs and reproduce but both males and females are feeding on plant sugars to supply carbohydrates, where they may acquire plant bacteria^3^. During the larval development of sand fly, they feed on detritus and other organic compounds, thus larvae expose high range of bacteria. The majority of these larval stage bacteria undergoes biodegradation during the pupal stage and the bacterial load decrease three days later following the emerge of an adult^4,5^. Female sand flies acquire *Leishmania* promastigotes from infected hosts and metacyclogenesis, a biological transformation from amastigote to promastigote, takes place in the midgut of the sand flies. During the metacyclogenesis, *Leishmania* resides permanently in the sand fly gut, thus possible bacteria-parasite interactions takes place between microbial community of the gut and parasite^6,7^.

There are several reports about the gut bacterial community of *Phlebotomus papatasi* but there is no data about the bacterial diversity of the Turkish sand fly populations^6,8,9^. The bacterial community is dependent to geographical distribution of sand fly and each geographically distinct population may have different bacterial compositions^10^. Bacterial diversity of the gut is reported to have significant effects on mosquitos, tsetse flies and sand flies^7,11,12^. Bacterial composition may either enhance or inhibit the parasitic activity and it’s strictly dependent to species of harboring bacteria. For instance, a bacterium present in the digestive tract of *P. duboscqi* has significant anti-parasitic effects on *Leishmania* development, while bacterial composition is critical factor for *Leishmania* growth in *Lutzomyia longipalpis* ^7,13^.

Due to this strong interaction between microbiome of the gut and the parasite, novel vector control approach called paratransgenesis, using genetically transformed microbes to express anti-parasitic molecules to reduce transmission, was applied in a previous study^14^. In this “Trojan Horse” approach, genetically modified transgenic bacteria are capable to express the desired anti-parasitic molecule, which could block the pathogen transmission in the vector species^12^. In order to apply paratransgenesis, first the suitable candidate bacteria should be identified in the vector species and host-bacteria interactions needs to be clarified for sustainability. The majority of the paratransgenic studies were conducted on mosquitos, triatominae bugs and tsetse flies^12,15^. However, limited studies were reported about the use of transformed bacteria on the control of leishmaniasis^14,16^.

Leishmaniasis is a significant public health problem in Turkey and cutaneous leishmaniasis (CL) is reported to be endemic in South eastern and Mediterranean Regions of Turkey^17^. Cutaneous leishmaniasis due to *Leishmania major* is recently reported in Turkey and 18 autochthonous cases were reported between 2011 and 2014^18^. *P. papatasi* is one of the most abundant species that reported in Turkey, which is the main vector of *L. major*, the causative agent of zoonotic cutaneous leishmaniasis (ZCL) endemic areas in Mediterranean Basin^18–20^.

The aim of this study was to identify the microbiome of different natural populations and endosymbionts of *P. papatasi* specimens collected in Turkey. We also aimed to identify the possible paratransgenic bacteria candidates, which could be used in the control of leishmaniasis in Turkey.

## Results

**Figure 1:**
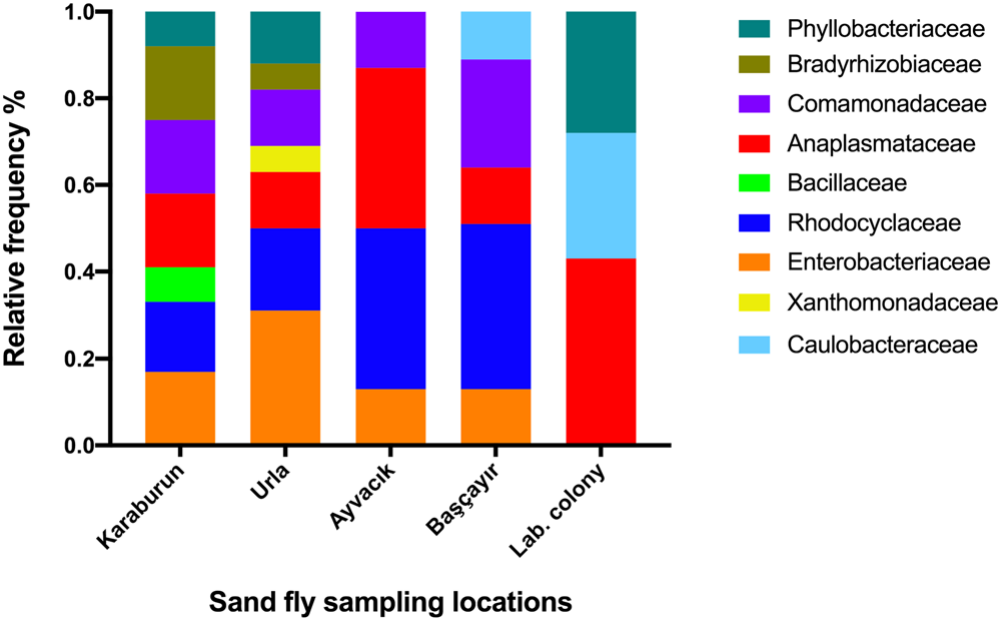
Relative abundance of gut bacteria of different *P. papatasi* populations. Relative gut bacteriome abundance shown in family level among different populations of *P. papatasi.*

**Figure 2:**
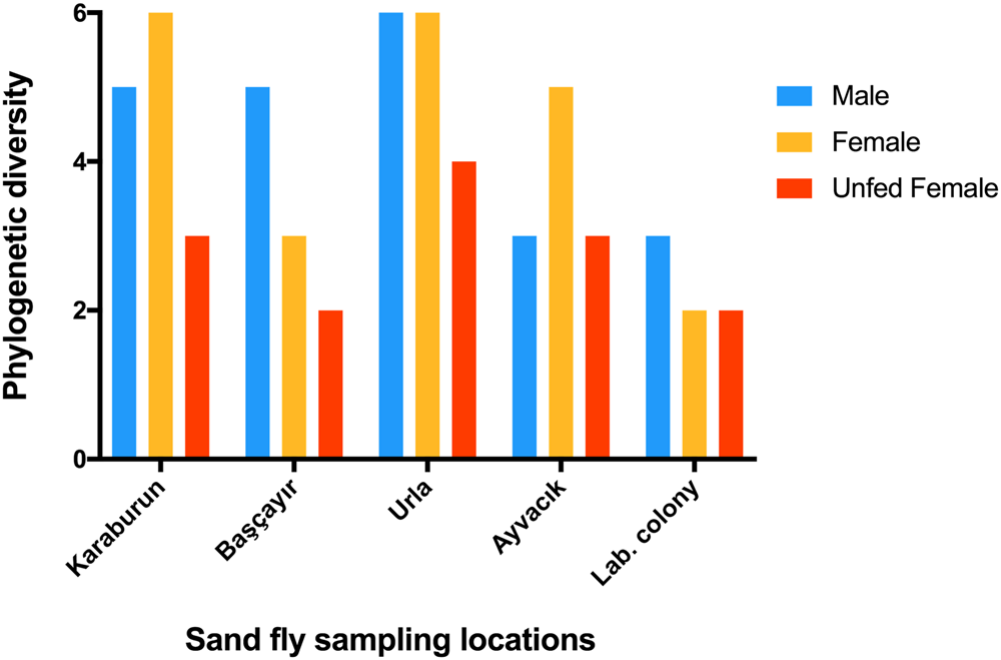
Phylogenetic diversity of gut bacteria of male, unfed female and blood-fed female of *P. papatasi* populations. Taxonomic diversity of different populations shown in genus level.

Totally, 120 field collected and 30 laboratory reared *P. papatasi* specimens were analyzed in the present study. Four different geographical groups (Karaburun, Urla, Başçayır and Ayvacik) and three sub-groups (blood-fed females, unfed females and males) were generated. Based on the sequence similarity to the previously submitted 16 S rRNA sequence data, 13 different bacteria (*Variovorax* sp., *Bosea* sp., *Brevundimonas* sp., *Ochrobactrum* sp., *Bacillus cereus, Erwinia aphidicola, Candidatus Ishikawaella capsulata*, *Klebsiella* sp., *Serratia marcescens, Stenotrophomonas maltophilia, Pantoea* sp., *Thauera* sp., and *Wolbachia* sp.) belonging to nine families (Anaplasmataceae, Bacillaceae, Bradyrhizobiaceae, Caulobacteraceae, Comamonadaceae, Enterobacteriaceae, Phyllobacteriaceae, Rhodocyclaceae, Xanthomonadaceae,) were identified from DGGE band sequences. Relative abundance (Fig. 1) of midgut and phylogenetic diversity (Fig. 2) among different populations are represented according to sequence data. Obtained sequence data were deposited to GenBank under the accession numbers (MF352020-MF352033).

All generated pools were screened for the presence of *Leishmania* spp. DNA by a real time PCR targeting ITS-1 region and no positivity was found.

### Microbiome profile of field collected *P. papatasi*

A total of 12 pools were generated using field collected *P. papatasi* and all groups were found positive for the presence of at least one bacteria. Among the field collected pools, highest diversity was observed in Urla (ten taxa) while the pool originated from Ayvacik were found to have lowest diversity (five taxa). Among the overall identified bacteria taxa, *Thauera* sp. (ten isolates) was the most frequent bacteria among the field collected specimens. One isolate was recorded for the following taxa *Bacillus cereus*, *Klebsiella* sp. *Serratia marcescens, Stenotrophomonas maltophilia* (Table 1).

**Table 1:** Microbiome profile of different *P. papatasi* populations.

Identified Bacteria	Karaburun Lat: 38.644410 Lon: 26.523768			Urla Lat: 38.245580 Lon: 26.683555			Ayvacik Lat: 39.650154 Lon: 26.305677			Başçayır Lat: 37.959298 Lon: 28.064445			Laboratory Colony		
	Male	Female	Blood-fed Female	Male	Female	Blood-fed Female	Male	Female	Blood-fed Female	Male	Female	Blood-fed Female	Male	Female	Blood-fed Female
*Variovorax sp*.	+	+		+	+			+			+	+			
*Bosea sp*.		+	+	+											
*Brevundimonas sp*.										+			+		+
*Ochrobactrum sp*.		+		+	+								+	+	
*Bacillus cereus*	+														
*Erwinia aphidicola*				+	+										
*Candidatus Ishikawaella capsulata*	+	+													
*Klebsiella sp*.					+										
*Serratia marcescens*								+							
*Stenotrophomonas maltophilia*				+											
*Alpha Proteobacterium*	+	+				+	+	+	+	+	+				
*Pantoea sp*.					+	+				+					
*Thauera sp*.	+		+	+	+	+	+	+	+	+	+	+			
*Wolbachia sp*.		+	+	+		+	+	+	+	+			+	+	+

### Microbiome profile of laboratory reared collected *P. papatasi*

Three taxa (*Brevundimonas* sp., *Wolbachia* sp. and *Ochrobactrum* sp.) were isolated from laboratory-reared colony. All study groups of laboratory colony were found positive for the presence of *Wolbachia* sp. in their guts. *Ochrobactrum* sp. was found in male and unfed female pool. Laboratory colony was found the lowest diverse (three taxa) group comparing to the field collected groups.

## Discussion

Midgut bacteriome and symbiosis in arthropods have been studied more than ever in recent years to identify the possible effects of bacteria on host-parasite interactions and their usage as a vector control agent. Four different *P. papatasi* populations and a laboratory reared colony were investigated in the present study and of the isolated bacteria, 13 out of 14 was gram negative. The predominancy of gram-negative bacteria in Diptera was reported previously^21^. Our results correlate with previous findings and one possible reasons of high gram negative prevalence in the sand fly midgut is due to the antimicrobial activity of gram negative bacteria against gram positive bacteria during the colonization^5,16,22,23^.

Sand flies are not free of bacteria since they emerged. Previous study reveals that newly emerged sand flies were associated with large amount of bacterial DNA, which could be taken from environment either by feeding or transstadial passage^4^. Most of the extracellular bacteria are unable to survive during the transstadial passage due to high antibacterial compound secretions in pupal stage but *Ochrobactrum* sp. is one of the transstadial bacteria, which is also known to be pathogenic to humans^5^. *Ochrobactrum* sp. was found only in the unfed female populations of Karaburun, while it is present in the male and unfed female populations of Urla and laboratory reared sand flies. Overall bacterial diversity was low in the laboratory colony generated pools, which was also reported in another study^23^. Such low diversity may be due to single diet regime applied in the laboratory colony during the sand fly colonization.

One of the major objectives of this study was to identify the possible control agents and their usage as a paratransgenic agent in the control of leishmaniasis in Turkey. Successful use of paratransgenic bacteria in sand flies was reported using *Bacillus subtilis* and the variants of *Bacillus* sp.^14^. The presence of *Bacillus* sp. in the natural population of Karaburun was demonstrated in the present study and its the only gram-positive bacteria identified in the present study. *Klebsiella* sp., *Pantoea* sp., and *Serratia* sp., was reported to be human pathogenic bacteria species and were identified in field collected sand flies in this study^24,25^.

Endosymbiont *Wolbachia* was reported in several studies including tsetse flies, mosquitos and also sand flies^7,26,27^. *Wolbachia* is a cytoplasmically inherited bacteria, which evolves with the host and the effects to the host is symbiotic rather that a parasitic^22^. High prevalence of *Wolbachia* sp. in mosquitos was previously noted in recent studies but there are limited reports on their presence in the sand flies^23,28,29^. In the present study *Wolbachia* sp. was the most prevalent bacteria among all pools and eleven isolate was reported. The use of *Wolbachia* sp. as vector control agent was performed in *Drosophila*, which kills the host by over-replication in the nervous system and shortens the average life span of the fly one and half comparing to uninfected flies^30^. Yet, there is no report has been published about use of *Wolbachia* sp. as a vector control agent in sand flies but the presence of this bacteria and transstadial passage to teneral flies makes *Wolbachia* sp. a possible control agent in sand flies.

*Variovorax s*p. is gram-negative bacteria, which is frequently isolated on contaminated waters and soil all around the world and one of the important plant growth promoting bacterium^31^. Presence of *Variovorax* sp. was reported on wild-caught *Anopheline* mosquitos in previous studies but this is the first report of its presence in the sand fly midgut^32^. Interestingly, *Variovorax* sp. was detected in all wild-caught populations in this study. At least one pool from each population was positive for *Variovorax* sp. but laboratory reared colony of *P. papatasi* was negative for *Variovorax* sp.

Pools consist of blood fed females was found to have the lowest bacterial prevalence in the present study. It is reported that bacterial richness was eventually decreased after blood feeding and gradually recovered in the following three days^7^. Comparing to blood fed sand flies, sucrose fed sand flies were reported to have highest bacterial prevalence^33^. Except Ayvacik population, bacterial diversity was found lower in all blood fed female pools. Pool containing unfed females was found to have two times higher bacterial richness comparing to blood fed and male pools. This would a possible result of a bacterial diversity decrease that take place after blood feeding. One possible reason for low bacterial diversity in Ayvacik male pool may be the feeding habit of the sand flies and limited sugar source for males in the study area.

Bacterial composition of the gut is mirrors the diet of sand fly^23^. Previous studies reported that bacteriome is more or less the reflection of the sand flies breeding site^16,25,34^. In the present study same populations have harbored the almost same bacterial diversity. Bacterial profile of male and unfed females are much similar comparing to blood fed females of the same population. This may be due to the changes of bacterial diversity right after blood feeding, which is also reported in previous studies^7^. The microbiome of the gut has also reported to effect the sand fly’s oviposition and larval development. Additionally, researchers reported that the bacterial richness of the habitat is an attractant for gravid *P. papatasi* in the field^33^.

Midgut is an important environment in the development of *Leishmania*, where the uninfective forms of *Leishmania* become infective. The possible effects of midgut bacteriome on the *Leishmania* development stages first suggested by Adler and Theodor^35^. The strong relation between bacteria, *Leishmania* and sand fly is reported in previous studies. There are contradictory reports about the interactions between sand fly microbiome and *Leishmania*. Antibiotic eradication of the midgut bacteriome resulted in inhibition of the growth of *Leishmania* ^7^. Researchers also reported that sand flies fed with sterile larval food resulted in high mortality rate after emerge^33^. Depending on the species, bacteria are proved to effect the *Leishmania* infection in sand flies either inhibiting or enhancing the transmission^4,7^. Bacterial composition of the sand fly midgut is also reported to change due to seasonal changes and climatic conditions^23^. Such changes in the midgut microbiome may be another factor that effects the transmission rate in endemic areas. Seasonal microbiome profiling studies in field collected sand flies will put forth valuable data in the control of sand flies by transgenic approach.

As a conclusion, microbiome of four different populations of *P. papatasi* was profiled in the present study. Presence of sand fly symbiont bacteria, *Wolbachia*, in natural populations of Turkey and possible control agents were also identified. Our next studies will aim the paratransgenic use of these identified bacteria as a vector control agent. This data presented here will provide other researchers a baseline support. We believe that further studies are needed to identify bacterial diversity of wild populations, thus bring the use of new possible symbionts in the vector control.

## Methods

### Sand fly rearing

For the maintenance of *P. papatasi* colony in the insectarium, methods suggested in a previous study were followed with a temperature of 26 ± 2 °C, a relative humidity between 60–70%, and a light:dark 14:10 h period^36^. Adult females were fed on anesthetized mice and both females and males were provided with 30% sucrose solution. Larval food was prepared as a mixture of rabbit feces and rabbit pellets without any sterilization step. Sand fly feeding process were carried out in accordance with relevant guidelines and regulations under the approval of Ethical Committee of Ege University 28.09.2011/05.

### Study area and Sand fly sampling

**Figure 3:**
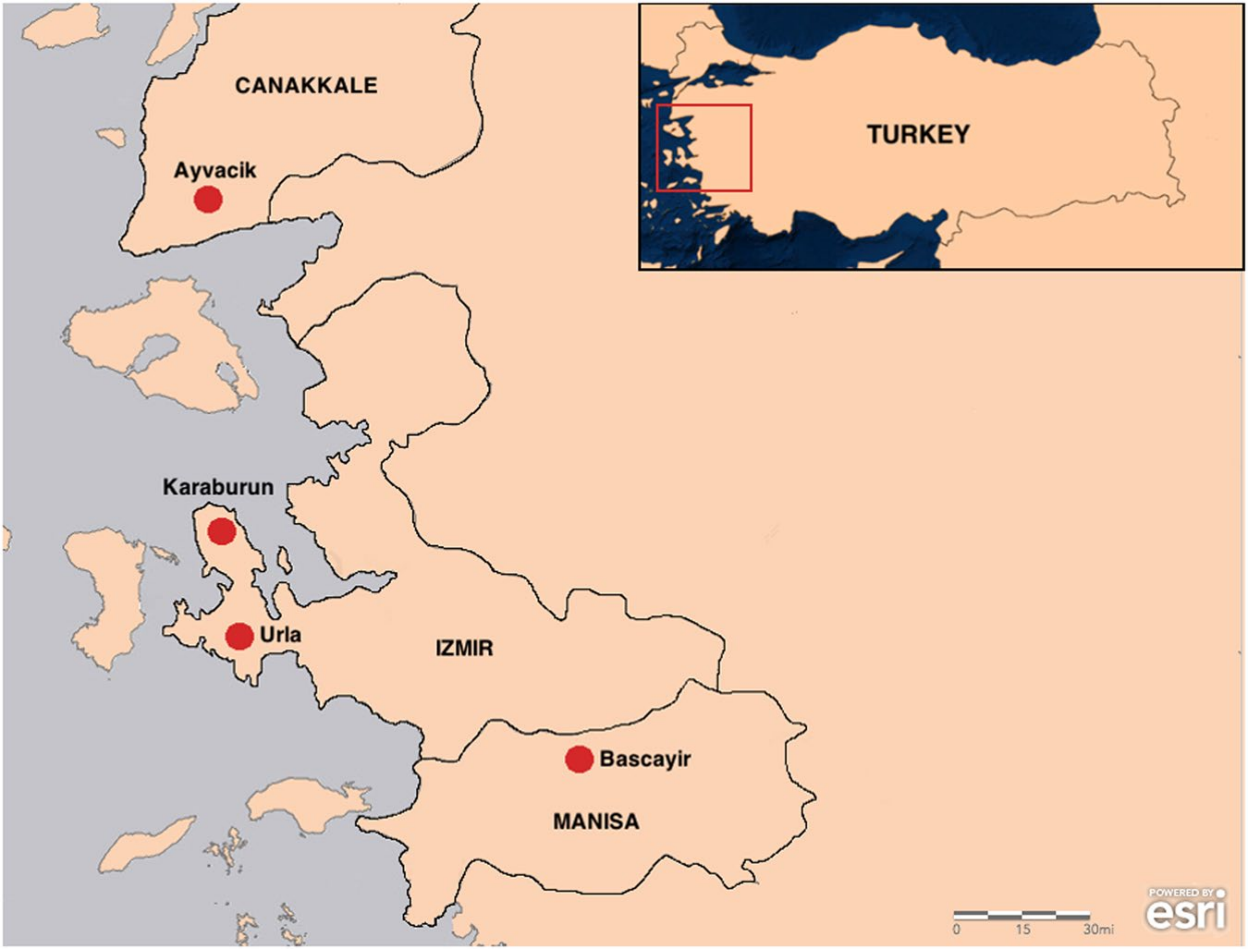
Sand fly collection sites. Map shown in this article was created using ArcGIS® software by Esri. ArcGIS® and ArcMap™ are the intellectual property of Esri and are used herein under license. Copyright © Esri. All rights reserved. For more information about Esri® software, please visit www.esri.com.

Sand flies were collected from four different towns (Karaburun, Urla, Başçayır and Ayvacik) located in western part of Turkey (Fig. 3).Study areas were determined according to previously reported CL cases and entomological records^17,18^. Study areas are located in Aegean region of Turkey, which has almost same vegetation. Sampling was done in two days for each collection site at the same time period (August 2014). The CDC miniature light traps were placed 100–120 cm above the ground level on animal shelters and gardens of the houses to obtain both blood fed female and male sand flies. Totally 20 light traps were set up in each village by the noon (6:00 pm) and collected following day before the sunrise (05:00 am). Wild-caught sand flies were transferred from traps to plexiglas carrying cages and moved to laboratory.

### Sand fly washing procedure and midgut dissection

Sand flies were immobilized on ice; and were divided into three groups for each population (blood-fed females, unfed females and males). In order to eliminate the bacterial contamination from cuticle, specimens were washed prior to dissections. **1**) Specimens were washed separately in micro centrifuge tubes with 100 μl PBS (Phosphate Buffered Saline) after this step **2**) each specimen washed in Antibiotic-Antimycotic (100X) solution then **3**) washed again with sterile molecular grade water. **4**) Sand flies were washed with 10% SDS solution and **5**) last washing was done using molecular grade water. **6**) Before midgut dissection step, sand flies were transferred to new sterile centrifuge tubes and gently vortexed.

After washing steps, sand flies were gently dissected under stereomicroscope using single use sterile insect needles. Dissections were done on sterilized single use slide covers and midguts were transferred to 1.5 ml centrifuge tubes after species identification. Pools were generated using 10 specimens for each study group. The head and genitalia of the sand flies were mounted on slide and species identifications were made using previously published written and electronic identification keys and descriptions for Mediterranean region^37–40^.

### DNA extraction and PCR

DNA extraction was made using Qiagen Blood&Tissue kit (Hilden, Germany) according to the manufacturers instructions. Nested PCR was applied to increase the sensitivity with different primer sets. In the first round all DNA samples were amplified with universal bacterial primers 27 F (5′-AGAGTTTGATCCTGGCTCAG-3′) and 1492r (5′-GGTTACCTTGTTACGACTT-3′)^41^. Amplifications were carried out using HelixAmpTM Taq Polymerase (Nanohelix, South Korea) and typical PCR mix contained 2.5 μl 10x reaction buffer, 0.5 μl dNTP mix (each 10 mM), 2 μl primer mix (each 10 μM), 0.625 U Taq DNA polymerase, 50 ng template DNA and ultrapure water to bring the final volume to 25 μl. PCR was performed with Techne TC-Plus thermal cycler (Bibby Scientific, Staffordshire, UK) using the following conditions; an initial denaturation step at 95 °C for 5 min, an amplification step 35 cycle of 95 °C for 20 sec, 55 °C for 40 sec, 72 °C for 90 sec, and final extension step at 72 °C for 5 min. PCR products were used as a template in the second amplification for denaturing gradient gel electrophoresis (DGGE) analysis with Bacterial 16 S rDNA V3 variable region primers 341F-GC (5′-GCCTACGGGAGGCAGCAG-3′ with GC clamps in its 5′ end) and 518 R (5′-ATTACCGCGGCTGCTGG-3′)^42^. PCR products were visualized after running on 1% agarose gel electrophoresis.

All generated pools were also studied, for detecting the presence of *Leishmania* DNA, by a real time PCR targeting ITS-1 region of *Leishmania* sp. using primers and probes as published previously^43^.

### Denaturing Gradient Gel Electrophoresis (DGGE)

DGGE was performed on DCode Universal Mutation System at 60 °C, 8% polyacrylamide gels with 35–60% denaturing gradient of urea-formamide (100% correspondent to 7 M urea and 40% [v/v] formamide). The electrophoresis conditions were 135 V in 1xTAE buffer for 8 hr. After DGGE, gel was stained with Biotium GelGreen dye (Hayward, CA) and bands were visualized under UVP Biospectrum Bioimaging Systems (Cambridge, UK). Separated DNA bands were cut with sterile scalpels and incubated in 100 µl sterile water at 4 °C for overnight. After incubation, 5 µl of the supernatant were used as template DNA in re-amplification reaction using the same primer sets (without GC-clamp) and reaction conditions described above. PCR products were sequenced commercially (MedSanTek, Istanbul).

### Sequencing and species typing

The sequence data obtained for the partial 16 S rRNA gene were aligned and analyzed using Geneious R9. Aligned sequence data was submitted to online 16 S Biodiversity tool, which is an automated BLAST (Basic Local Alignment Search Tool) search tool and able to analyze multiple sequence data at same time^44^. Nucleotide similarity score threshold was set to be higher than 95% and sequences were identified both on genus and species level according to their similarity scores. Additionally; sequence data was submitted to GenBank and accession numbers were recorded.

### Data availability

The datasets generated and analyzed during the current study are available from the corresponding author on reasonable request.

## Electronic supplementary material

Dataset 1
